# Retinoic acid promotes differentiation of WiT49‐ but not of CCG99‐11 Wilms tumour cells

**DOI:** 10.1002/cnr2.1819

**Published:** 2023-04-25

**Authors:** Caroline Jansson, Linda Holmquist Mengelbier

**Affiliations:** ^1^ Division of Clinical Genetics, Department of Laboratory Medicine Lund University Sweden

**Keywords:** CCG99‐11, differentiation, model system, retinoic acid, Wilms tumour, WiT49

## Abstract

**Background:**

Most children with Wilms tumour are successfully treated with multidrug chemotherapy and surgery. These treatments cause severe side effects for the patients, an issue that needs to be addressed by exploring other treatment options with less or no side effects. One option is to complement current therapies with agents that could potentially induce tumour cell differentiation, for example retinoic acid (RA).

**Aims:**

To facilitate quick assessment of an agent's effect on Wilms tumour differentiation by a rapid in vitro model system.

**Methods and Results:**

Here WiT49 and CCG99‐11 Wilms tumour cells were treated with 10 μM RA for 72 h or 9 days. Cultured cells were scraped off from Petri dishes, pelleted and embedded in paraffin in the same way as clinical tumour specimens are preserved. Cell morphology and differentiation were evaluated by analyses of haematoxylin eosin (H&E) and immunohistochemical stainings. Based on H&E, WT1 and CKAE1/3 stainings, RA treatment induced further epithelial differentiation of WiT49 cells, whereas there was no sign of induced maturation in CCG99‐11 cells. Ki67 staining showed that RA inhibited cell proliferation in both cell lines.

**Conclusions:**

Our study shows that in vitro culturing of WiT49 and CCG99‐11 cells, followed by pelleting and paraffin embedding of cell pellets, could aid in a quick evaluation of potential differentiating agents against Wilms tumour. In addition, our results strengthen previous results that retinoic acid could be a potential complement to regular Wilms tumour treatment.

## 1 INTRODUCTION

Wilms tumour is a paediatric solid tumour of the kidney. Most patients with Wilms tumour have a good prognosis thanks to multidrug chemotherapy and surgery, sometimes supplemented by radiotherapy. However, the treatment often comes with lifelong side effects.^1^ To increase the quality of life for Wilms tumour survivors, therapies need to be modulated with agents less harmful than chemotherapy. Such agents might not always kill off the cancer cells, but rather inhibit their proliferation and induce a less malignant phenotype, that is, induce the cancer cells to differentiate. The strategy of differentiation therapy in cancer treatment is to induce terminal differentiation of the cancer cells.^2^

Retinoic acid (RA), a metabolite of vitamin A, which cause only mild side effects when distributed as a drug, has been used rather successfully as part of the treatment for patients afflicted with leukaemia and neuroblastoma.^3^ Neuroblastoma is a solid paediatric tumour derived from the sympathetic nervous system and differentiation of neuroblastoma cells can be analysed in vitro. For instance, differentiation of neuroblastoma derived cell lines can be induced by retinoic acid and is recognised by increased neurite outgrowths (reviewed in).^4^ It is less clear how to evaluate differentiation of Wilms tumour cells in vitro.

Cultures with primary Wilms tumours have been established as 3D spheroids or organoids and are perfect for exploration of patient‐specific drug sensitivities.^5^, ^6^, ^7^ Even so, Wilms tumour is a rare disease, the number of patients and thus the extent of clinical tumour material is limited. There is a need for an alternative way to make pilot testing of drugs before irreplaceable primary cultures are exhausted. Here, established Wilms tumour cell lines become important.

Wilms tumours are known for their commonly triphasic histology consisting of blastema, stroma and epithelium.^8^ Those histological components can to some extent be recreated by cell line based orthotopic Wilms tumour xenograft models and differentiation status can be evaluated microscopically.^9^, ^10^, ^11^ Drawbacks with xenograft models are the aspects of animal ethics and time. Therefore, research on cell lines would benefit from a more ethical and less time consuming perspective to do pilot testing of potential differentiation inducing agents.

The differentiated epithelial component of Wilms tumour is identified by the presence of epithelial tubules. Accordingly, a procedure to evaluate Wilms tumour differentiation in vitro would require the possibility to analyse the formation of epithelial tubules, along with assessment of a panel of differentiation markers. Thus, the aim of this study is to establish whether Wilms tumour cell lines cultured in vitro can be used to evaluate a drug's capacity to induce differentiation of Wilms tumour cells. RA is explored as differentiation agent.

Two well established cell lines that are uniformly recognised as Wilms tumour derived cells, are WiT49 and CCG99‐11.^12^, ^13^ Our research group have earlier characterised them as epithelial and blastemal predominant cell lines respectively.^9^, ^11^ In order to assess the differentiation status, knowledge about the origin of Wilms tumour is necessary.

Wilms tumour mimics the developing kidney, nephrogenesis, with respect to its three distinct histological components described above. Nephrogenesis is initiated by the reciprocal induction between the metanephric mesenchyme, that is, the blastema, and the ureteric bud. Upon induction of nephrogenesis a mesenchymal to epithelial transition takes place and blastema differentiates into mature epithelial tubular structures. SIX2 is expressed in the immature blastema of the developing kidney and is found to be expressed in Wilms tumour blastema. WT1, on the other hand, is important at several stages of nephrogenesis and nuclear expression is frequently recognised in Wilms tumour cells.^14^, ^15^ Mature epithelial tumour tubules can be highlighted by expression of specific keratins.^16^

Experimentally, RA has been shown to be important in kidney development.^17^ In a zebrafish nephrogenesis model systems it has been shown that RA plays a central role in differentiation and segmentation of the nephrons.^18^ Previous studies of RA and Wilms tumour show that RA can inhibit proliferation of primary Wilms tumour cultures and create morphological changes.^19^, ^20^ On the transcriptional level, it has been demonstrated that RA treatment of cultured Wilms tumour cells enhances gene expression of RA‐ and TGF‐β signalling related genes, which are genes involved in inhibition of cell proliferation. It has also been shown that RA mitigates the expression of genes associated with high risk and relapse.^19^, ^20^ The number of patients with Wilms tumour that have been enroled in clinical trials with RA as treatment agent is limited.^21^, ^22^

This study shows how the WiT49 and CCG99‐11 cell lines can be used as an in vitro model system to evaluate the differentiation status of cultured Wilms tumour cells. In vitro cultured cells were pelleted and paraffin embedded, and differentiation status was analysed by haematoxylin eosin staining (H&E) and, at the protein level, by immunohistochemistry. With this model system the induction of Wilms tumour differentiation by RA treatment could be determined. RA induced further epithelial differentiation of WiT49 cells, whereas this was not apparent for CCG99‐11 cells. RA treatment inhibited proliferation of both cell lines.

## 2 MATERIALS AND METHODS

### 2.1 Cell culture and retinoic acid treatment

WiT49 and CCG99‐11 cells were cultured in DMEM F:12 (1:1) medium (ThermoFisher Scientific) supplemented with 1% PenStrep and 5% or 10% foetal bovine serum (HyClone FBS, Nordic Biolabs). Cells were confirmed to be mycoplasma free by qPCR based testing provided by Eurofins Genomics. The phenotypes of the cell lines were in line with those established in our previous paper confirming no contamination from any other cell line population.^11^ Cells were seeded at day 0 and treated with either DMSO (as a control) or RA (ATRA R2625, Merck). Experiments started the next day when the cells had adhered. Cells were cultured for either 72 h or long term (LT) cultured for 9 days. For 72 h experiments, cells were treated with 10 μM RA on day 1 only. LT cultured cells were initially seeded into 60 mm cell culture dishes and upon confluency, cells were trypsinated and expanded into larger cell culture dishes (100 mm, followed by 150 mm). For LT experiments cells were treated with 10 μM RA at day 1 and in conjunction with expansion of the cells into larger cell culture dishes at days 4 and 7. Harvest took place on day 10, corresponding to 9 days of treatment. Except for dead cells floating in the media, no cells were discarded during the experiment.

At the end of the experiments, cells were gathered by either trypsination or cell scraper and washed with 1× PBS twice followed by pelleting in order to create paraffin embedded cell blocks. Cell pellets were treated with 4% paraformaldehyde for 20 min, followed by incubation with haematoxylin for 5 min; 70% ethanol (EtOH) o.n.; 95% EtOH for 1 h; 99,5% EtOH for 1 h; xylen for 1 h, followed by paraffin embedding at 65°C for at least 1 h. All steps were performed at room temperature unless indicated, and all cell pelleting steps between treatments were done by centrifugation for 5 min at 300g.

### 2.2 Experimental set up

Three 72 h experiments were performed for each cell line, where the cells from the first experiment was harvested by trypsination and the following two by scraping off the cells from the cell culture dishes (WiT49: n = 3; CCG99‐11: n = 3). For long term experiments: 2 experimental replicates were performed for each cell line; LT 5% FBS (WiT49: n = 2; CCG99‐11: n = 2) and LT 10% (WiT49: n = 2; CCG99‐11: n = 2). All IHC markers used in this study have been applied to all harvested cell pellets, although only representative pictures are shown.

### 2.3 Immunohistochemistry on cell pellets

On positively charged slides, 4 μm thick sections (FFPE) were dried for 20 min in 60°C. The slides were deparaffinised in Xylene and rehydrated in ethanol to water. Heat Induced Epitope Retrieval (HIER) was carried out by incubation of tissue sections in Target Retrieval Buffer pH 9.0 (WT1 and SIX2; S2367, DAKO), or pH 6.0 (CKAE1_2 and Ki67; S1699, DAKO) and 0,2% Triton X‐100 (T8787‐50ML, Sigma), in a Decloaking Chamber (NxGen, Biocare Medical, CA) for 20 min in 95°C. Endogenous peroxidase was blocked for 20 min with 1% H_2_O_2_ (95321‐100ML, Sigma–Aldrich) diluted in PBS pH 7.4 (A9201, 0010, Applichem). The sections were incubated with primary antibodies against WT1 (WT1‐RTU, clone 6F‐H2, DAKO), CKAE1_3 (MAB3412, Millipore), Ki67 (M7240, DAKO) and SIX2 (66347‐1‐lg). Except for WT1, which was prediluted, all primary antibodies were diluted in PBS containing 5% normal goat serum (005‐000‐001, Jackson Immuno Research). Immunostainings were visualised using BrightVision Poly‐HRP‐ Anti mouse RTU (DPVR55HRP, AH Diagnostics) for 30 min, followed by incubation with DAB (Liquid DAB+ Substrate Chromogen System, K3477, DAKO) for 5 min, and counterstained with Mayers Htx (01820, Histolab) for 30 s. All incubations were performed at room temperature and sections were washed three times with PBS after each incubation. Slides were mounted with Faramount Aqueous Mounting Medium (S3025, DAKO).

### 2.4 Proliferation index based on Ki67

WiT49 and CCG99‐11 cells grown with 10% FBS and treated with either RA (10 μM) or DMSO were cultured for either 72 h (n = 3 for each cell line) or 9 days (n = 2 for each cell line). For 72 h experiments, both cells that had been trypsinated and scraped off upon harvest were included in the analysis. Cell pellets were immunohistochemically stained with Ki67 (see above) and images were acquired at 10× magnification. The Ki67 index was determined by the total number of Ki67 positive cells/total number of cells in each image. For details about data analysis, see ‘Data and statistical analyses’ below.

### 2.5 Data and statistical analyses

Ki67 stained nuclei were counted in cells cultured at 10% FBS and treated with either DMSO or RA. All three 72 h CCG99‐11 experiments (DMSO vs. RA) were analysed (experiments with cells collected by trypsination, n = 1; experiments with cells collected by scraping, n = 2) together and all three 72 h WiT49 experiments were analysed (collected by trypsination, n = 1; cells collected by scraping, n = 2) together. The DMSO 72 h group cells were compared to the RA 72 h treated group cells (experiments n = 3). Two LT (DMSO or RA treated) experiments were analysed for CCG99‐11 and WiT49 cells. For each cell line (WiT49 and CCG99‐11), the LT DMSO cells were compared to the LT RA cells (experiments n = 2). To obtain a robust number of cell nuclei to analyse, all cell nuclei within one 10× magnification image/treatment/experiment were counted followed by the number of cell nuclei positive for Ki67. Thus, the total number of cells analysed from each experimental set up was as follows: 72 h CCG99‐11 cells: DMSO (n = 8875) and RA (n = 9278); 72 h WiT9 cells: DMSO (n = 4216) and RA (n = 3584); LT CCG99‐11 cells: DMSO (n = 6236) and RA (n = 5842); LT WiT49 cells: DMSO (n = 3312) and RA (n = 1176). Fisher's exact test (two‐sided) was used to determine any statistical differences between groups. If the number of scored cells were plenty/too many for a Fisher's exact test to be performed, a Chi‐square test with Yates correction was carried out (CCG99‐11 cells). Both tests were performed through the online Graph Pad software (https://www.graphpad.com/quickcalcs/contingency2/). Any *p*‐value <.01 was considered significant.

## 3 RESULTS

### 3.1 Scraped off Wilms tumour cells facilitate the study of epithelial structures in vitro

**FIGURE 1:**
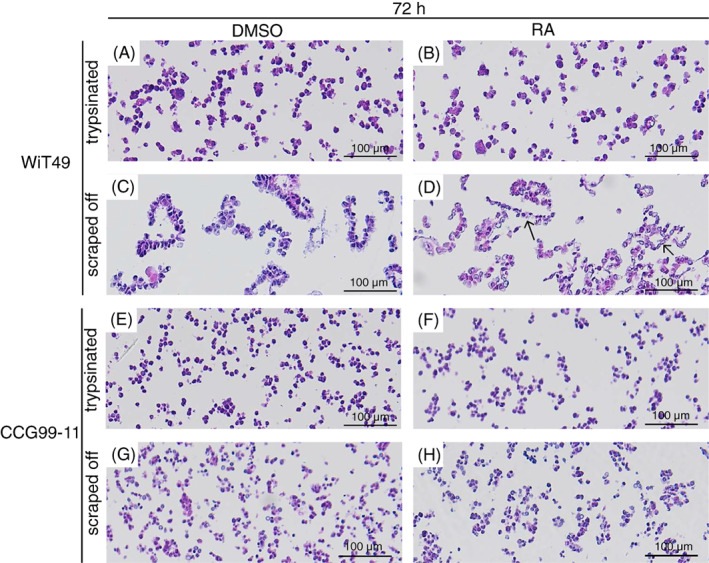
Retinoic acid induces morphological changes of WiT49 Wilms tumour cells. Heamatoxylin eosin stains of WiT49 (A–C) and CCG99‐11 (E–H) cells, treated with either DMSO or retinoic acid (RA) for 72 h. Cells were either collected by trypsination (A, B, E and F) or scraped off with a cell scraper (C, D, G and H). Cells were treated with 10 μM RA. The experiments were repeated twice for scraped off and once for trypsinated cells. Cells were treated with 10 μM RA. Representative pictures are shown. Arrows in 1D point at thin garland formations.

Initially, the task was to establish in what way to collect cultured WiT49 and CCG99‐11 Wilms tumour cells to enable an easy way to study epithelial structures in vitro. After 72 h's growth in Petri dishes, cells were either trypsinated or scraped off, then pelleted and embedded in paraffin. After applying the cell scraper technique, WiT49 cells were shown to grow in condensed sheets of cells, which mimics the growth of epithelial/tubular structures (Figure 1C,D). Scraped off CCG99‐11 cells did show adherence between cells, but there was no indication of growth of epithelial structures (Figure 1G,H). As expected, it was not possible to study the morphology of trypsinized cells as such cells all appear rounded (Figure 1A,B,E,F). Thus, for assessment of cell morphology, only cells which had been collected using a cell scraper were analysed, since that technique enabled sheets of cells to stay more intact (Figure 1C,D,G,H).

### 3.2 Retinoic acid induced differentiation of WiT49 cells

To test the effect of RA on Wilms tumour cell differentiation, WiT49 and CCG99‐11 cells were treated with 10 μM RA, or DMSO as a control, for 72 h. WiT49 cells treated with RA for 72 h were more elongated than control cells as the RA treated cells lined up in thin pronounced garland formations, mirroring segmentation during nephrogenesis, which is a sign of epithelial differentiation (Figure 1C,D). RA treatment of CCG99‐11 cells did not induce any apparent morphological changes (Figure 1G,H).

**FIGURE 2:**
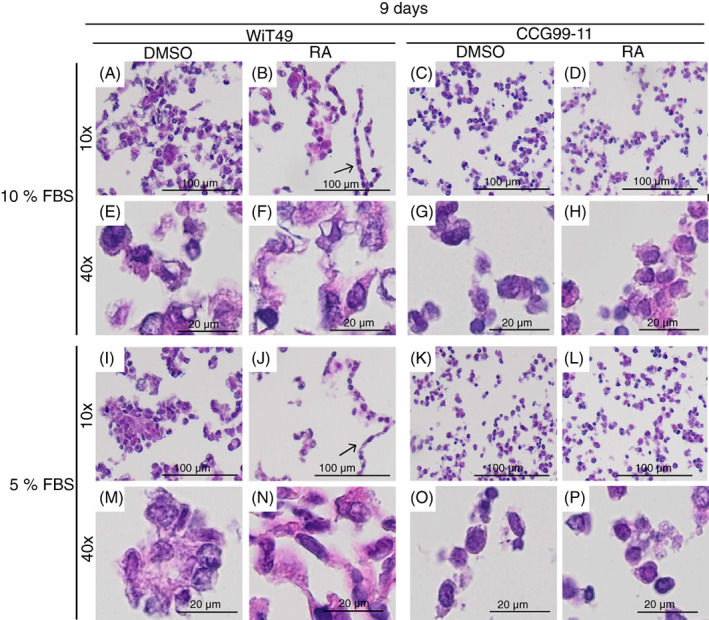
Prolonged retinoic acid treatment of WiT49 and CCG99‐11 cells enhances morphological changes whereas reduced serum levels do not. Heamatoxylin eosin stains of WiT49 and CCG99‐11 cells treated by retinoic acid (RA) for 9 days in 10 (A–H) or 5% (I–P) FBS. Pictures from the same experiment were taken at both 10× and 40× magnifications. Cells were treated with 10 μM RA. Experiments with prolonged RA treatment were repeated twice at 5% and twice at 10% FBS. Representative pictures are shown from one 5% and one 10% FBS experiment. Arrows in 2B and 2J point at thin garland formations.

To test if a longer RA treatment could induce signs of differentiation of CCG99‐11 cells, the treatment was prolonged from 3 to 9 days. RA kept WiT49 cells in a morphologically more differentiated state also after 9 days of RA treatment, with even more pronounced garland formations than after 72 h RA treatment (Figure 2A,B,E,F, cf. Figure 1C,D). Despite prolonged RA treatment of CCG99‐11 cells, RA did still not induce differentiation of CCG99‐11 cells (Figure. 2C,D,G,H cf. Figure 1G,H). Taken together, prolonged RA treatment accentuated the RA induced differentiation of WiT49 cells whereas no signs of cellular maturation were detected in RA treated CCG99‐11 cells.

### 3.3 Reduced serum levels do not induce differentiation of CCG99‐11 cells

To elucidate if serum levels could aid in RA induced differentiation of CCG99‐11 cells, the cell culture serum levels were reduced from 10% to 5% FBS for both control (DMSO treated) and RA treated cells. Despite reduced serum levels, there were no signs of any RA effects on CCG99‐11 cell morphology (Figure 2K,L,O,P). The differentiation effect of RA on WiT49 cells, under reduced serum levels, was equal to that at 10% FBS (Figure 2I,J,M,N). As the initial evaluation of differentiation status, based on morphology, were similar if cells were treated with either 5% or 10% serum for 9 days, both these experimental set ups were evaluated in further aspects of differentiation.

### 3.4 WT1 protein/nuclear expression is affected by prolonged retinoic acid treatment

**FIGURE 3:**
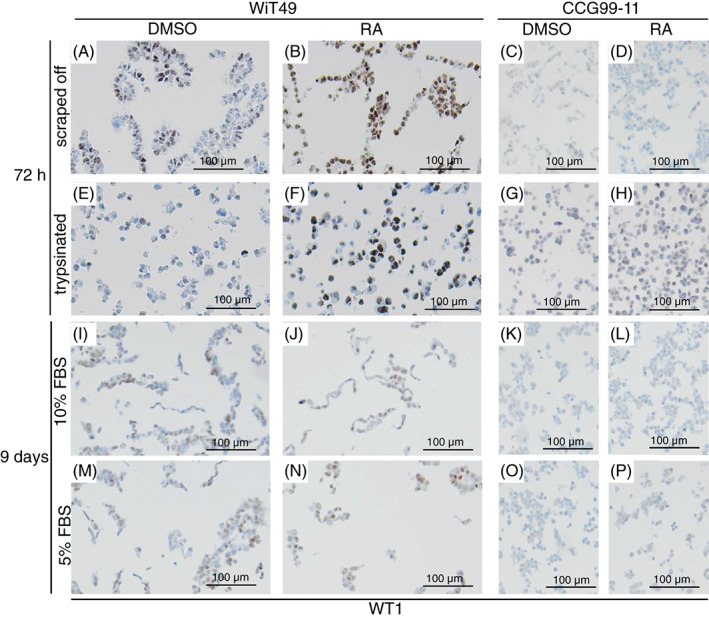
Nuclear WT1 expression is distinct in WiT49 cells upon 72 h retinoic acid treatment, but fades after 9 days of treatment. Immunohistochemically detected WT1 expression in scraped off (A–D) or trypsinated WiT49 and CCG99‐11 cells (E–H) after 72 h DMSO or retinoic acid (RA) treatment. Cells treated with 10 μM RA. WT1 expression in WiT49 and CCG99‐11 cells after 9 days of RA treatment in cells cultured at either 10 (I–L) or 5% FBS (M–P). Representative pictures are shown. All panels showing CCG99‐11 cells are also shown in extended formats in Figure S2.

WT1 is known to act during the induction of mammalian nephrogenesis, but its expression is also known to fluctuate over time during different stages of kidney development.^14^, ^23^ It is known to be expressed in Wilms tumour.^14^ After 72 h RA treatment, nuclear WT1 expression became more accentuated and intense in WiT49 cells (Figure 3A,B), which was even more clear when looking at the trypsinated cells (Figure 3E,F). Nuclear WT1 expression appeared equivalent in RA treated and control (DMSO treated) WiT49 cells after 9 days of treatment (Figure 3I,J,M,N). WT1 protein was not induced by RA in CCG99‐11 cells after neither 72 h (Figure 3C,D,G,H; Figure S2A–D), nor 9 days of treatment (Figure 3K,L,O,P; Figure S2E–H).

### 3.5 Epithelial markers cytokeratin1_3 are augmented by retinoic acid in WiT49 cells

**FIGURE 4:**
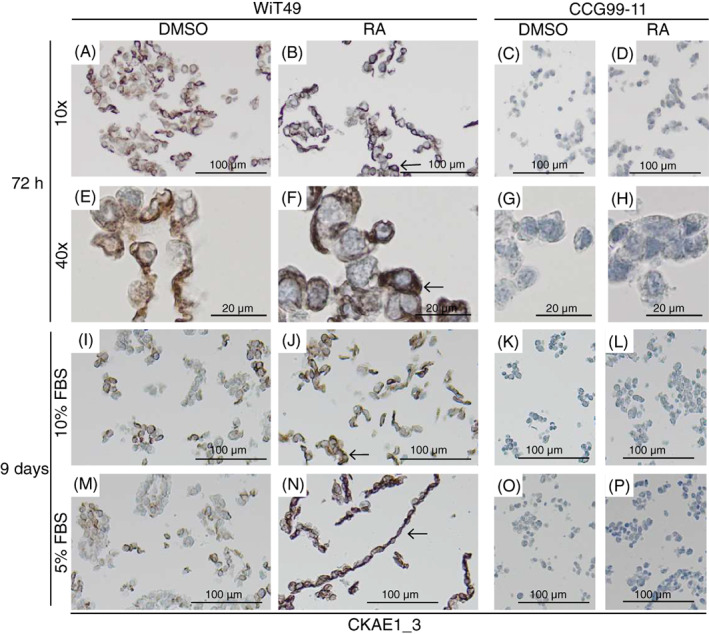
Retinoic acid induces strong CKAE1_3 expression in WiT49 cells – a sign of increased differentiation. WiT49 (A, B, E and F) and CCG99‐11 (C, D, G and H) cells cultured in 10% FBS and treated with retinoic acid (RA), or DMSO as control, for 72 h, or for 9 days at either 10% (I–J) or 5% FBS (M–P). Cells treated with 10 μM RA. Representative pictures are shown. Arrows in 4B, 4F, 4 J and 4 N point at strong membranous CKAE1_3 positivity. Annotations 10× and 40×, indicate which microscopic magnification was used when the pictures were taken.

Keratins are expressed by epithelial cells and used to define epithelial differentiation of Wilms tumours. CKAE1_3 corresponds to a cocktail of keratins, that is, cytokeratin1–8, 10, 14–16 and 19, but does not detect CK17 or CK18. CKAE1_3 membrane positivity was detected in the epithelial‐like cell line WiT49 irrespective of RA treatment, but its expression was weaker and less organised in DMSO treated control cells as opposed to RA treated WiT49 cells where CKAE1_3 membranous positivity was prominent and well organised after 72 h (Figure 4A,B,E,F). The RA induced enhanced epithelial maturation seen in WiT49 cells persisted after 9 days of RA treatment (Figure 4I,J,M,N). This suggests that RA treatment induces further maturation of WiT49 cells. The blastemal‐like CCG99‐11 cells did not express CKAE1_3 at all (Figure 4C,D,G,H; Figure S3A–D) not even after prolonged RA treatment (Figure 4K,L,O,P; Figure S3E–H).

### 3.6 SIX2 – a marker of immature cells – is not expressed in either WiT49 or CCG99‐11 cells

**FIGURE 5:**
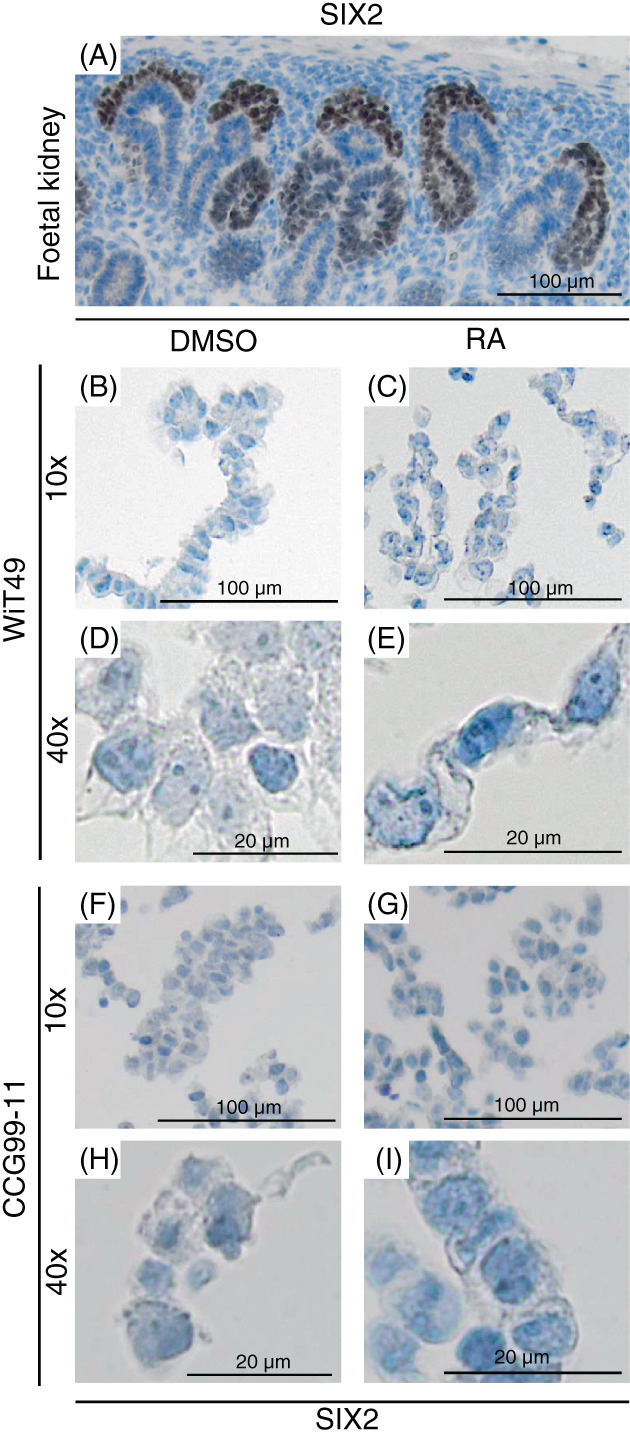
The blastemal marker SIX2 is not expressed in either WiT49 or CCG99‐11 cells. (A) Cap mesenchyme and comma shaped bodies in human foetal kidney are stained with SIX2 (DAB, brown). (B–I) SIX2 in WiT49 (B–E) and CCG99‐11 (F–I) cells treated with either DMSO, as a control, or RA for 72 h. Pictures from the same experiments were taken at both 10× and 40× magnifications.

SIX2 is expressed in the metanephric mesenchyme of the human foetal kidney (Figure 5A) and it is usually found to be expressed in Wilms tumour blastema.^24^, ^25^ If RA induces differentiation of Wilms tumour cells, in theory, decreased SIX2 expression should be noted in RA treated cells. However, none of the cell lines stained positive for SIX2, whether treated with DMSO or RA (Figure 5B–I). Thus, this marker of immaturity could not give any indication of induced differentiation in either CCG99‐11 or WiT49 cells.

### 3.7 Retinoic acid inhibits cell proliferation in both WiT49 and CCG99‐11 cells

Upon cell maturation, cell proliferation is usually inhibited. Cells treated with RA at 10% FBS for either 72 h or 9 days were stained with the proliferation marker Ki67 (Figure S1). The ratio of Ki67 positive cells were significantly lower in both WiT49 and CCG99‐11 cells treated with RA in comparison to control cells treated with DMSO only (CCG99‐11 72 h: *p* < .0001, 9 days: *p* < .001, Chi‐square with Yates correction, two tailed and WiT49 72 h: *p* < .0001, 9 day: *p* < .0001 Fisher's exact test, two tailed). In summary, RA treatment significantly inhibited cell proliferation in both WiT49 and CCG99‐11 cells.

## 4 DISCUSSION

Although the survival rate of children diagnosed with Wilms tumour is good, treatment with heavy chemotherapy mostly cause lifelong adverse effects.^1^ To reduce the adverse effects of present treatment strategies, introduction of drugs with more gentle side effects is to be preferred. Differentiation therapy, which aims to terminally differentiate malignant cancer cells into their original benign cells, is one way to tackle this concern. In addition, if researchers turn to already existing drugs to use for drug repurposing, then there is already some knowledge about treatment side effects. To explore new, or repurposed drugs, for differentiation therapy of patients with Wilms tumour, without draining clinical samples or performing unnecessary animal experiments, basic researchers would benefit from an uncomplicated method to screen drugs.

In the present study established Wilms tumour cell lines have been in vitro cultured, pelleted and analysed by H&E and immunohistochemistry, in line with histological analysis of Wilms tumours at a clinical pathology department. The choice to scrape off cells from the culture dish enabled more ‘histology’‐like analyses of the monolayers of cells than when loosening cells by trypsination. When harvesting the cells by cell scraper instead of by trypsination, cell morphology was preserved and epithelial structures could be distinguished. The method is straightforward to perform. However, tumour heterogeneity, captured by primary Wilms tumour organoids and 3D cultures of primary blastemal Wilms tumours, is not properly represented here as the present system is based on cell lines.^6^, ^7^

The response to RA in the two different cell lines were disparate, as only WiT49 showed a clear induction of epithelial differentiation by distinct garland formations. Why some Wilms tumour cells will be susceptible to induction of differentiation and others not remain to be elucidated. When cells are not induced to differentiate upon treatment this could be due to the fact that cells are cultured as monolayers without any added matrix to interact with, which could be a confounding factor when a cell line, like CCG99‐11 do not form epithelial structures upon treatment but another one (WiT49) do. Analyses of additional Wilms tumour cell lines would be beneficial. Unfortunately, there are few established Wilms tumour cell lines. Especially since over the years some of them have been re‐evaluated and been designated as other tumour entities based on for instance their genetic profiles.^26^, ^27^, ^28^

Tumour genetics could also explain why the two cell lines respond differently. *SIX2* is recurrently mutated in Wilms tumour, but mutations in the gene do not seem to affect SIX2 protein expression according to the literature.^29^ To our knowledge nobody has compiled information about if mutated *SIX2* renders Wilms tumour cells unable to differentiate. It has previously been shown that the blastemal marker SIX2 is not expressed at the protein level in WiT49 cells, which is strengthened here.^12^ Based on orthotopic xeonograft tumours CCG99‐11 has previously been classified with blastemal like characteristics, including a slight SIX1 expression.^11^ SIX2 is commonly expressed in Wilms tumour blastema and it could be expected that SIX2 was expressed in CCG99‐11 cells, but it was not in the cell cultures of the present study. To resolve whether wild type SIX2 expression is necessary or not for induction of differentiation of Wilms tumour cells is out of scope for this study but remains to be established.

WT1, is a periodic/fluctuating actor in nephrogenesis.^30^ Nuclear WT1 was induced in WiT49 cells after 72 h RA treatment, whereas this increase in WT1 expression was not visible after prolonged RA treatment (9 days), which could be related to prolonged enhanced differentiation. Could *WT1* status affect the response to RA? The Gessler group have previously treated primary Wilms tumour cells from two different patients; one tumour had predominantly stromal histology and a *WT1* mutation, whereas the other tumour consisted of primary cells from a triphasic tumour without *WT1* mutation. Both responded to RA by increasing gene expression of RA‐signalling genes and reverted the gene expression of genes related to high risk and relapsed Wilms tumours.^19^ WiT49 cells express wild type *WT1*,^12^ whereas the WT1 status of CCG99‐11 is to our knowledge unknown.

The inhibition of proliferation induced by RA in WiT49 and CCG99‐11 cells in our study is in line with what has been shown for primary Wilms tumour cultures.^19^, ^20^ Wegert *et al* also showed that RA effects were reversible when discontinuing the treatment of the cells.^20^ Such results might be discouraging, but RA could potentially be a not so toxic drug that could be used as metronomic cancer treatment.^31^ There is an assumed positive effect of RA treatment of patients with Wilms tumour, however, the number of patients that have received RA therapy is too small to give conclusive results.^21^, ^22^ Our results support further evaluation of the use of RA in treatment of children with Wilms tumour. Most importantly, our method for evaluation of induced differentiation of Wilms tumour cell lines can be used in future studies in the search for agents, such as for instance natural compounds, which are known to only cause mild side effects to the patients.^32^

## 5 CONCLUSIONS

In this work, the fact that WiT49 cells are epithelial and CCG99‐11cells are blastemal in their characteristics are strengthen.^11^ It is also shown that by harvesting cells by scraping them off, instead of by trypsination, before pelleting the cells, cell culture dish cell morphology is well preserved, enabling in vitro evaluation of epithelial differentiation of Wilms tumour cell lines. By use of the simple in vitro system described above, it could be established that RA induces further epithelialisation of WiT49 cells. The model system could be used for screening other potential differentiation inducing agents. This will be an aid in Wilms tumour research, which aims to ameliorate the quality of life for children that have survived Wilms tumour.

## AUTHOR CONTRIBUTIONS

**Caroline Jansson:** Methodology (equal); writing – original draft (supporting); writing – review and editing (supporting). **Linda Holmquist Mengelbier:** Conceptualization (lead); data curation (equal); formal analysis (lead); funding acquisition (lead); investigation (lead); methodology (equal); project administration (lead); supervision (lead); visualization (lead); writing – original draft (lead); writing – review and editing (lead).

## FUNDING INFORMATION

The study was supported by grants from the Royal Physiographic Society, Sweden, and iCOPE (the interregional Childhood Oncology Precision medicine Exploration).

## CONFLICT OF INTEREST STATEMENT

The authors have stated explicitly that there are no conflicts of interest in connection with this article.

## ETHICS STATEMENT

This work was performed on established Wilms tumour cell lines.

## Supporting information


**Supplemental Figure S1.** Retinoic acid inhibits proliferation of both WiT49 and CCG99‐11 cells. WiT49 and CCG99‐11 cells treated with 10 μM retinoic acid (RA) for either 72 h (A–H) or 9 days (I–L). Cells were gathered by either trypsination (A–D) or scraped off (E–L) and cell pellets stained with Ki67. The pictures are representative snippets of the 10× images that were used to calculate Ki67 indexes.

**Supplemental Figure S2.** Lack of WT1 expression in CCG99‐11 cells. Cells were treated with DMSO (control) or RA according to the outline in Figure 3. That is, cells were treated with 10 μM retinoic acid (RA) or DMSO for 72 h or 9 days. The 9 day experiments were performed at either 5 or 10% FBS. Panel A–H are the same, but extended, images as in Figure 3C,D,G,H,K,L,O,P. Representative pictures are shown.

**Supplemental Figure S3.** Lack of CKAE1_3 expression in CCG99‐11 cells. Cells were treated with DMSO (control) or retinoic acid (RA) according to the outline in Figure 4. That is, cells were treated with 10 μM RA or DMSO for 72 h or 9 days. The 9 day experiments were performed at either 5 or 10% FBS. Panel A–H are the same, but extended, images as in Figure 3C,D,G,H,K,L,O,P. Representative pictures are shown.

## Data Availability

Data sharing not applicable ‐ no new data generated.

## References

[1] Spreafico F , Fernandez CV , Brok J , et al. Wilms tumour. Nat Rev Dis Primers. 2021;7(1):75. 34650095 10.1038/s41572-021-00308-8

[2] de Thé H . Differentiation therapy revisited. Nat Rev Cancer. 2018;18(2):117‐127. 29192213 10.1038/nrc.2017.103

[3] Masetti R , Biagi C , Zama D , et al. Retinoids in pediatric onco‐hematology: the model of acute promyelocytic leukemia and neuroblastoma. Adv Ther. 2012;29(9):747‐762. 22941525 10.1007/s12325-012-0047-3

[4] Bayeva N , Coll E , Piskareva O . Differentiating Neuroblastoma: a systematic review of the retinoic acid, its derivatives, and synergistic interactions. J Pers Med. 2021;11(3):211. 10.3390/jpm11030211 PMC7999600 33809565

[5] Wegert J , Bausenwein S , Roth S , Graf N , Geissinger E , Gessler M . Characterization of primary Wilms tumor cultures as an in vitro model. Genes Chromosomes Cancer. 2012;51(1):92‐104. 22034155 10.1002/gcc.20936

[6] Wegert J , Zauter L , Appenzeller S , et al. High‐risk blastemal Wilms tumor can be modeled by 3D spheroid cultures in vitro. Oncogene. 2020;39(4):849‐861. 31562394 10.1038/s41388-019-1027-8 PMC6976522

[7] Calandrini C , Schutgens F , Oka R , et al. An organoid biobank for childhood kidney cancers that captures disease and tissue heterogeneity. Nat Commun. 2020;11(1):1310. 32161258 10.1038/s41467-020-15155-6 PMC7066173

[8] Vujanić GM , Parsons LN , D'Hooghe E , Treece AL , Collini P , Perlman EJ . Pathology of Wilms' tumour in International Society of Paediatric Oncology (SIOP) and Children's oncology group (COG) renal tumour studies: similarities and differences. Histopathology. 2022;80(7):1026‐1037. 35275409 10.1111/his.14632

[9] Holmquist Mengelbier L , Lindell‐Munther S , Yasui H , et al. The Iroquois homeobox proteins IRX3 and IRX5 have distinct roles in Wilms tumour development and human nephrogenesis. J Pathol. 2019;247(1):86‐98. 30246301 10.1002/path.5171 PMC6588170

[10] Bielen A , Box G , Perryman L , et al. Dependence of Wilms tumor cells on signaling through insulin‐like growth factor 1 in an orthotopic xenograft model targetable by specific receptor inhibition. Proc Natl Acad Sci USA. 2012;109(20):E1267‐E1276. 22529373 10.1073/pnas.1105034109 PMC3356645

[11] Mengelbier LH , Bexell D , Sehic D , Ciornei CD , Gisselsson D . Orthotopic Wilms tumor xenografts derived from cell lines reflect limited aspects of tumor morphology and clinical characteristics. Pediatr Blood Cancer. 2014;61(11):1949‐1954. 25044705 10.1002/pbc.25131

[12] Alami J , Williams BR , Yeger H . Derivation and characterization of a Wilms' tumour cell line, WiT 49. Int J Cancer. 2003;107(3):365‐374. 14506735 10.1002/ijc.11429

[13] Kim MK , Mason JM , Li CM , et al. A pathologic link between Wilms tumor suppressor gene, WT1, and IFI16. Neoplasia. 2008;10(1):69‐78. 18231640 10.1593/neo.07869 PMC2213901

[14] Goyal S , Mishra K , Sarkar U , Sharma S , Kumari A . Diagnostic utility of Wilms' tumour‐1 protein (WT‐1) immunostaining in paediatric renal tumours. Indian J Med Res. 2016;143(Supplement):S59‐s67. 27748279 10.4103/0971-5916.191776 PMC5080930

[15] Hohenstein P , Pritchard‐Jones K , Charlton J . The yin and yang of kidney development and Wilms' tumors. Genes Dev. 2015;29(5):467‐482. 25737276 10.1101/gad.256396.114 PMC4358399

[16] Sredni ST , Neves JI , de Camargo B , Caballero OL , Soares FA . Pan‐cytokeratin immunoexpression in Wilms' tumors: a simple approach for understanding tumor epithelial differentiation. Sao Paulo Med J. 2004;122(4):181‐183.1554337610.1590/s1516-31802004000400011

[17] Takayama M , Miyatake K , Nishida E . Identification and characterization of retinoic acid‐responsive genes in mouse kidney development. Genes Cells. 2014;19(8):637‐649. 24962468 10.1111/gtc.12163

[18] Wingert RA , Selleck R , Yu J , et al. The cdx genes and retinoic acid control the positioning and segmentation of the zebrafish pronephros. PLoS Genet. 2007;3(10):1922‐1938. 17953490 10.1371/journal.pgen.0030189 PMC2042002

[19] Zirn B , Samans B , Spangenberg C , Graf N , Eilers M , Gessler M . All‐trans retinoic acid treatment of Wilms tumor cells reverses expression of genes associated with high risk and relapse in vivo. Oncogene. 2005;24(33):5246‐5251. 15897880 10.1038/sj.onc.1208725

[20] Wegert J , Bausenwein S , Kneitz S , et al. Retinoic acid pathway activity in Wilms tumors and characterization of biological responses in vitro. Mol Cancer. 2011;10:136. 22067876 10.1186/1476-4598-10-136 PMC3239322

[21] Adamson PC , Matthay KK , O'Brien M , Reaman GH , Sato JK , Balis FM . A phase 2 trial of all‐trans‐retinoic acid in combination with interferon‐alpha2a in children with recurrent neuroblastoma or Wilms tumor: a pediatric oncology branch, NCI and Children's oncology group study. Pediatr Blood Cancer. 2007;49(5):661‐665. 16900483 10.1002/pbc.21011

[22] Friesenbichler W , Krizmanich W , Lakatos K , et al. Outcome of two patients with bilateral nephroblastomatosis/Wilms tumour treated with an add‐on 13‐cis retinoic acid therapy – case report. Pediatr Hematol Oncol. 2018;35(3):218‐224. 30260265 10.1080/08880018.2018.1515284

[23] Li H , Hohenstein P , Kuure S . Embryonic kidney development, stem cells and the origin of Wilms tumor. Genes (Basel). 2021;12(2):318. doi:10.3390/genes12020318 PMC7926385 33672414

[24] Pierce J , Murphy AJ , Panzer A , et al. SIX2 effects on Wilms tumor biology. Transl Oncol. 2014;7(6):800‐811. 25500091 10.1016/j.tranon.2014.09.005 PMC4311027

[25] Murphy AJ , Pierce J , de Caestecker C , et al. SIX2 and CITED1, markers of nephronic progenitor self‐renewal, remain active in primitive elements of Wilms' tumor. J Pediatr Surg. 2012;47(6):1239‐1249. 22703800 10.1016/j.jpedsurg.2012.03.034 PMC3377935

[26] Stroup EK , Yeu Y , Budhipramono A , et al. WT‐CLS1 is a rhabdoid tumor cell line and can be inhibited by miR‐16. Cancer Rep (Hoboken). 2019;2(3):e1110.

[27] Smith MA , Morton CL , Phelps D , Girtman K , Neale G , Houghton PJ . SK‐NEP‐1 and Rh1 are Ewing family tumor lines. Pediatr Blood Cancer. 2008;50(3):703‐706. 17154184 10.1002/pbc.21099

[28] Pritchard‐Jones K , Perotti D . WARNING: G‐401 and SK‐NEP‐1 cell lines are not Wilms tumor cell lines. Pediatr Blood Cancer. 2019;66(7):e27741. doi:10.1002/pbc.27741 30924592

[29] Wegert J , Ishaque N , Vardapour R , et al. Mutations in the SIX1/2 pathway and the DROSHA/DGCR8 miRNA microprocessor complex underlie high‐risk blastemal type Wilms tumors. Cancer Cell. 2015;27(2):298‐311. 25670083 10.1016/j.ccell.2015.01.002

[30] Rivera MN , Haber DA . Wilms' tumour: connecting tumorigenesis and organ development in the kidney. Nat Rev Cancer. 2005;5(9):699‐712. 16110318 10.1038/nrc1696

[31] Pramanik R , Bakhshi S . Metronomic therapy in pediatric oncology: a snapshot. Pediatr Blood Cancer. 2019;66(9):e27811. 31207063 10.1002/pbc.27811

[32] Mijatović S , Bramanti A , Nicoletti F , Fagone P , Kaluđerović GN , Maksimović‐Ivanić D . Naturally occurring compounds in differentiation based therapy of cancer. Biotechnol Adv. 2018;36(6):1622‐1632. 29656090 10.1016/j.biotechadv.2018.04.001

