# Supplementary figures and images for: Retinoic acid promotes differentiation of WiT49‐ but not of CCG99‐11 Wilms tumour cells
Source: Cancer Rep (Hoboken). 2023 Apr 25;6(6):e1819. doi: 10.1002/cnr2.1819 (PMC10242656; doi:10.1002/cnr2.1819)

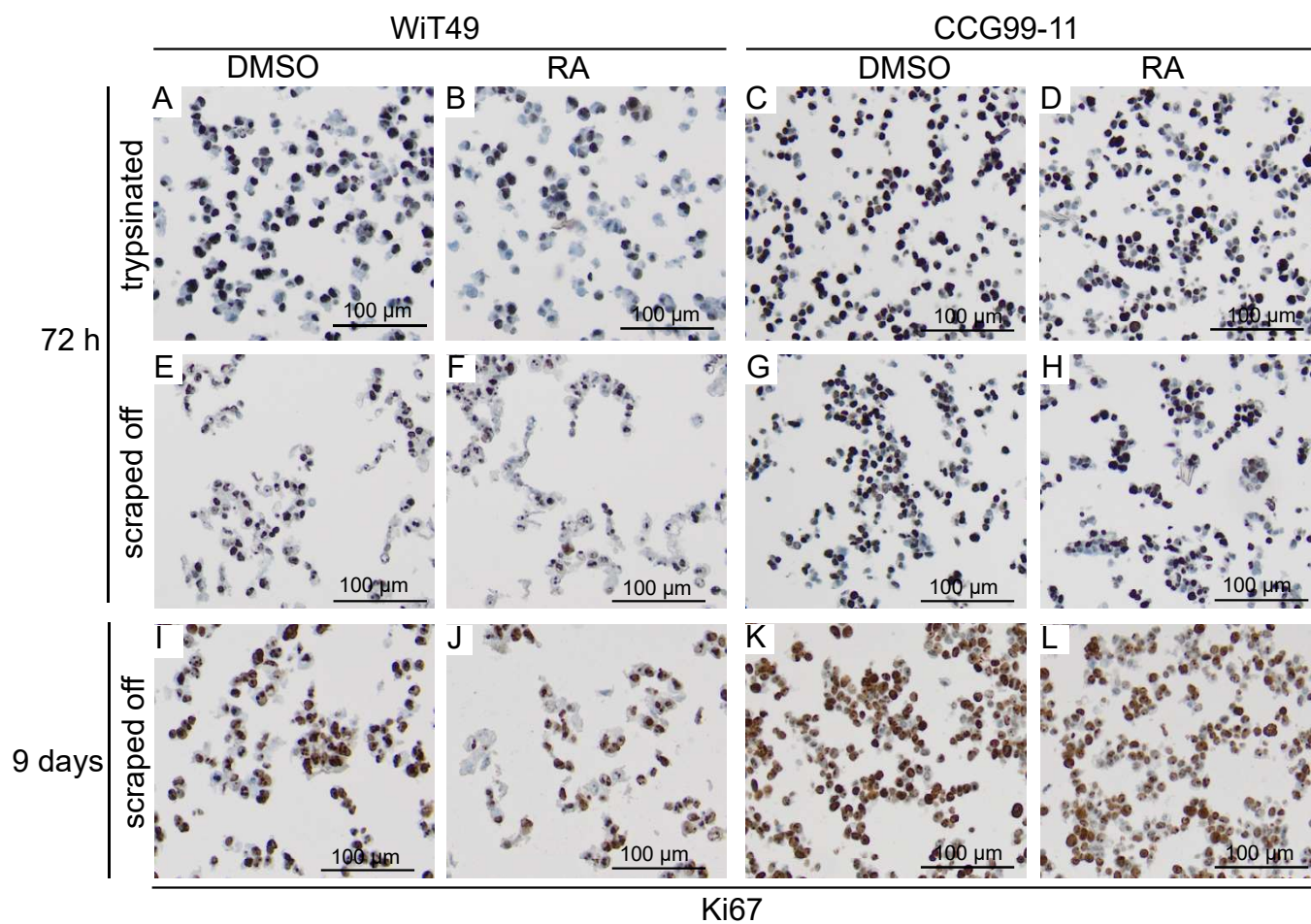

Supplement: Supplementary file 1 — Supplemental Figure S1. Retinoic acid inhibits proliferation of both WiT49 and CCG99‐11 cells. WiT49 and CCG99‐11 cells treated with 10 μM retinoic acid (RA) for either 72 h (A–H) or 9 days (I–L). Cells were gathered by either trypsination (A–D) or scraped off (E–L) and cell pellets stained with Ki67. The pictures are representative snippets of the 10× images that were used to calculate Ki67 indexes. [file CNR2-6-e1819-s003.pdf]

CCG99-11

DMSO

RA

72 h

scraped off

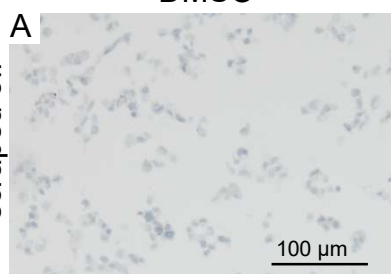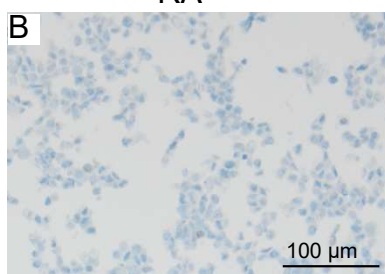

trypsinated

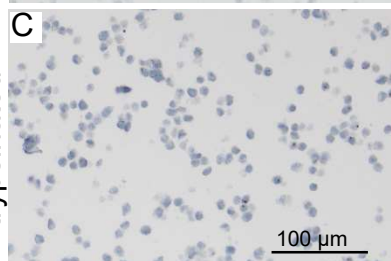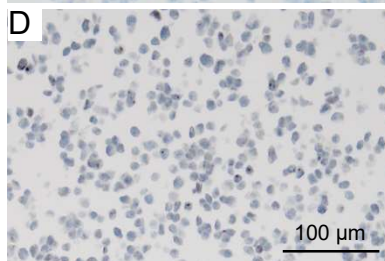

9 days

10% FBS

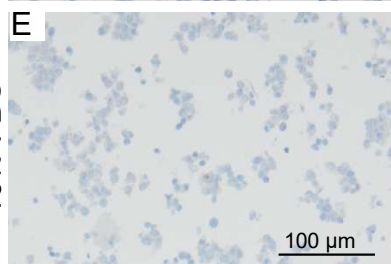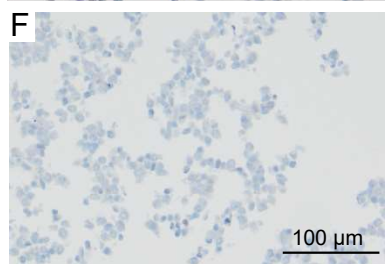

5% FBS

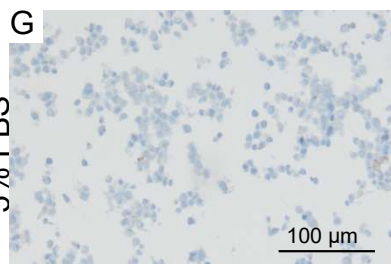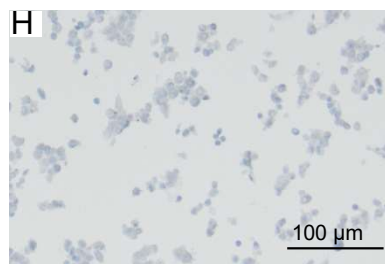

WT1

Supplement: Supplementary file 2 — Supplemental Figure S2. Lack of WT1 expression in CCG99‐11 cells. Cells were treated with DMSO (control) or RA according to the outline in Figure 3. That is, cells were treated with 10 μM retinoic acid (RA) or DMSO for 72 h or 9 days. The 9 day experiments were performed at either 5 or 10% FBS. Panel A–H are the same, but extended, images as in Figure 3C,D,G,H,K,L,O,P. Representative pictures are shown. [file CNR2-6-e1819-s001.pdf]

CCG99-11

DMSO

RA

10x

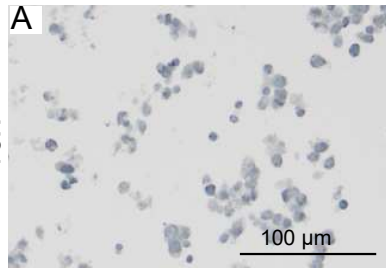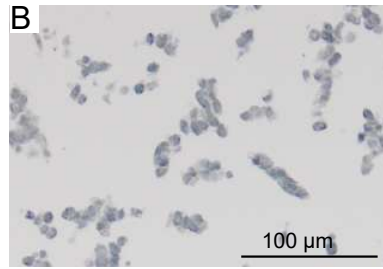

72 h

40x

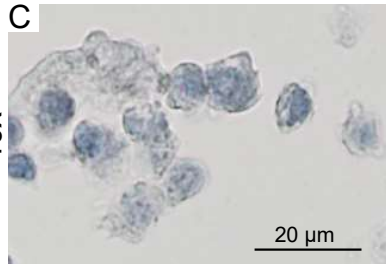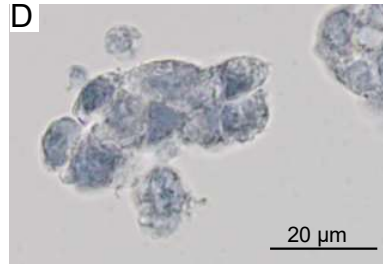

10% FBS

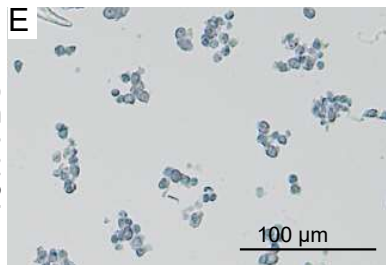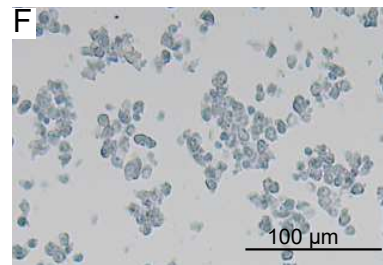

9 days

5% FBS

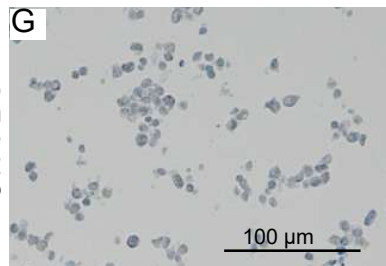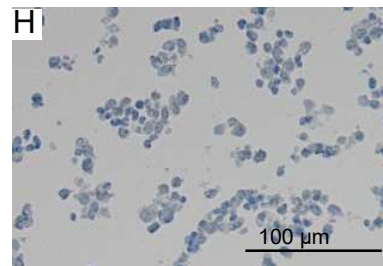

CKAE1\_3

Supplement: Supplementary file 3 — Supplemental Figure S3. Lack of CKAE1_3 expression in CCG99‐11 cells. Cells were treated with DMSO (control) or retinoic acid (RA) according to the outline in Figure 4. That is, cells were treated with 10 μM RA or DMSO for 72 h or 9 days. The 9 day experiments were performed at either 5 or 10% FBS. Panel A–H are the same, but extended, images as in Figure 3C,D,G,H,K,L,O,P. Representative pictures are shown. [file CNR2-6-e1819-s002.pdf]
